# Supplementary material for: Involvement of miR-190b in Xbp1 mRNA Splicing upon Tocotrienol Treatment
Source: Molecules. 2020 Dec 31;26(1):163. doi: 10.3390/molecules26010163 (PMC7795057; doi:10.3390/molecules26010163)

Supplementary Figure S1. Effects of γT3 (20 µg/mL for 24 h), δT3 (10 µg/mL for 24 h) in HeLa cell transfected with pre-miR-190b (pre190b) and anti-miR-190b (an190b) on mRNA expression of Xbp1. BFA (2.5 µg/mL for 8 h) was used as inducer of EndoR stress. Results are reported as mean ± SD of data obtained from 9 independent experiments. Bars show log_2 Fold Change (treated vs control). CC, control cells. Data were analysed by Kruskal-Wallis test. *p*-values ≤ 0.05 were considered statistically significant.

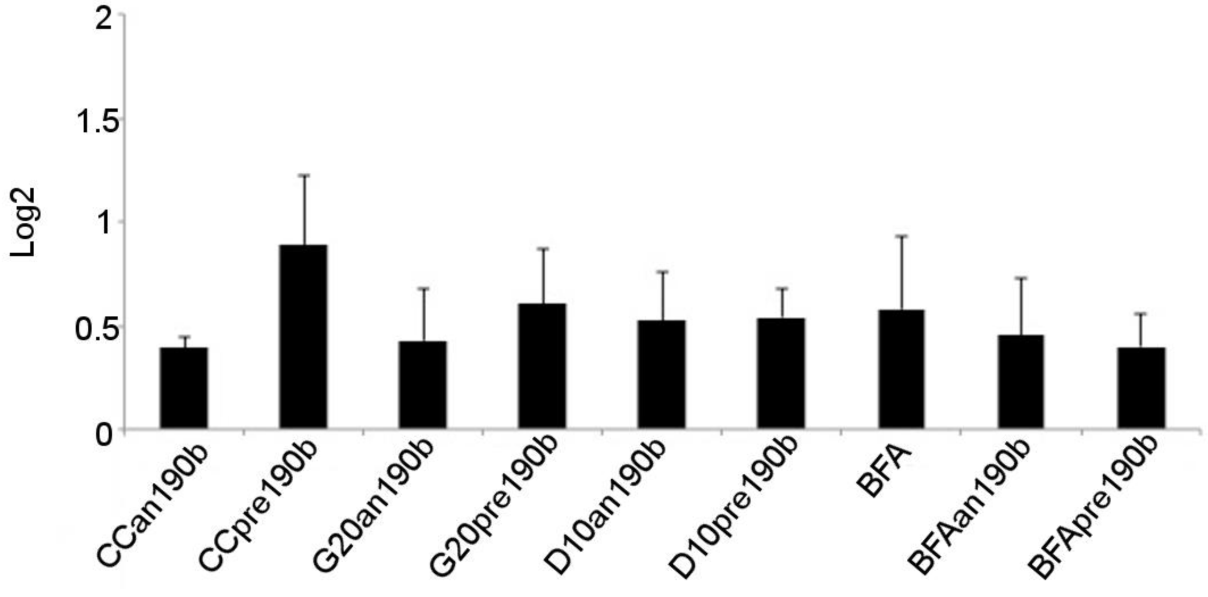

Supplementary Figure S2. Validation of cellular model. Effect of γT3 (20 µg/mL for 24 h) and δT3 (10 µg/mL for 24 h) on miR-190b expression by RT-PCR, in HeLa cell transfected with pre-miR-190b (pre190b) and anti-miR-190b (an190b). TUN (5 µg/mL for 4 h) and BFA (2.5 µg/mL for 8 h) were used as inducer of EndoR stress. The figure shows data obtained from 5 independent experiments. Bars show log 2 Fold Change (treated vs control). CC, control cells. Data were analysed by one-way ANOVA with repeated measures followed Tukey's test. Different letters indicate significant differences ($p < 0.05$)

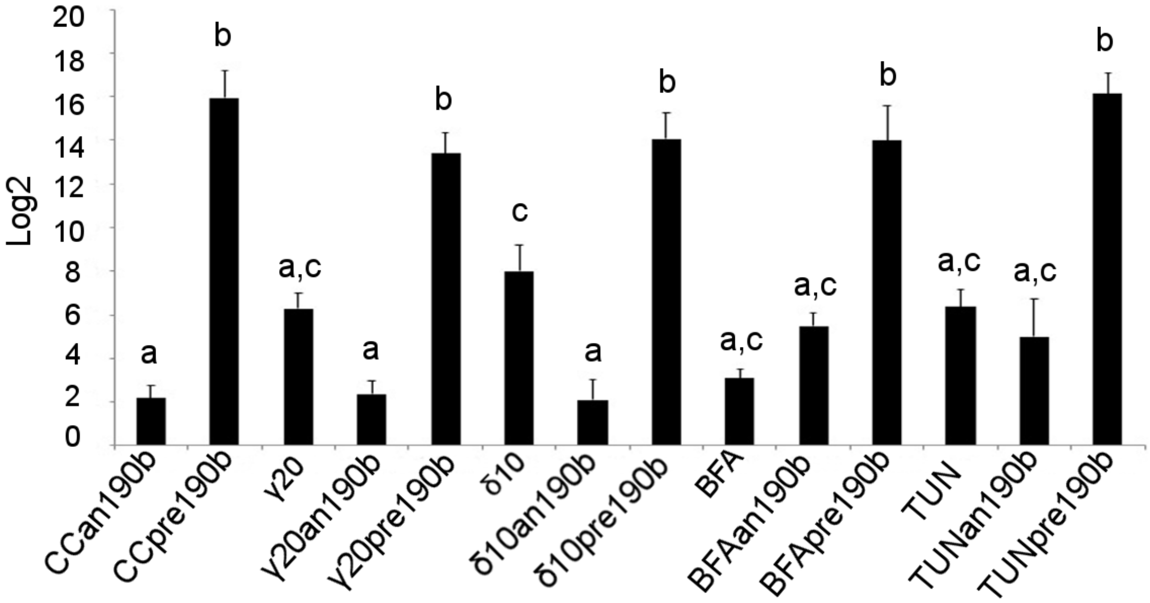

Supplement: Supplementary file 1 [file molecules-26-00163-s001.pdf]
